# Matching Biomedical Ontologies: Construction of Matching Clues and Systematic Evaluation of Different Combinations of Matchers

**DOI:** 10.2196/28212

**Published:** 2021-08-19

**Authors:** Peng Wang, Yunyan Hu, Shaochen Bai, Shiyi Zou

**Affiliations:** 1 School of Computer Science and Engineering Southeast University Nanjing China; 2 School of Artificial Intelligence Southeast University Nanjing China; 3 Southeast University - Monash University Joint Graduate School Suzhou China

**Keywords:** biomedical ontology, ontology matching, matching clues, reduction anchors

## Abstract

**Background:**

Ontology matching seeks to find semantic correspondences between ontologies. With an increasing number of biomedical ontologies being developed independently, matching these ontologies to solve the interoperability problem has become a critical task in biomedical applications. However, some challenges remain. First, extracting and constructing matching clues from biomedical ontologies is a nontrivial problem. Second, it is unknown whether there are dominant matchers while matching biomedical ontologies. Finally, ontology matching also suffers from computational complexity owing to the large-scale sizes of biomedical ontologies.

**Objective:**

To investigate the effectiveness of matching clues and composite match approaches, this paper presents a spectrum of matchers with different combination strategies and empirically studies their influence on matching biomedical ontologies. Besides, extended reduction anchors are introduced to effectively decrease the time complexity while matching large biomedical ontologies.

**Methods:**

In this paper, atomic and composite matching clues are first constructed in 4 dimensions: terminology, structure, external knowledge, and representation learning. Then, a spectrum of matchers based on a flexible combination of atomic clues are designed and utilized to comprehensively study the effectiveness. Besides, we carry out a systematic comparative evaluation of different combinations of matchers. Finally, extended reduction anchor is proposed to significantly alleviate the time complexity for matching large-scale biomedical ontologies.

**Results:**

Experimental results show that considering distinguishable matching clues in biomedical ontologies leads to a substantial improvement in all available information. Besides, incorporating different types of matchers with reliability results in a marked improvement, which is comparative to the state-of-the-art methods. The dominant matchers achieve F1 measures of 0.9271, 0.8218, and 0.5 on Anatomy, FMA-NCI (Foundation Model of Anatomy-National Cancer Institute), and FMA-SNOMED data sets, respectively. Extended reduction anchor is able to solve the scalability problem of matching large biomedical ontologies. It achieves a significant reduction in time complexity with little loss of F1 measure at the same time, with a 0.21% decrease on the Anatomy data set and 0.84% decrease on the FMA-NCI data set, but with a 2.65% increase on the FMA-SNOMED data set.

**Conclusions:**

This paper systematically analyzes and compares the effectiveness of different matching clues, matchers, and combination strategies. Multiple empirical studies demonstrate that distinguishing clues have significant implications for matching biomedical ontologies. In contrast to the matchers with single clue, those combining multiple clues exhibit more stable and accurate performance. In addition, our results provide evidence that the approach based on extended reduction anchors performs well for large ontology matching tasks, demonstrating an effective solution for the problem.

## Introduction

### Background

In recent years, various biomedical ontologies, such as National Cancer Institute (NCI) Thesaurus [1], Foundation Model of Anatomy (FMA) [2], Systemized Nomenclature of Medicine (SNOMED-Clinical Terms [SNOMED-CT]) [3], have been widely used in various fields, such as for medical data formats standardization [4], medical or clinical knowledge representation and integration [5], and medical decision making [6]. With the continuous evolution of biomedical data, biomedical terminology is characterized by complexity and ambiguity, which further complicates intelligent biomedical applications. Furthermore, emerging biomedical ontologies are built independently, with various ways of defining same biomedical components, resulting in heterogeneous problems. To implement the interoperability across biomedical ontologies, the establishment of meaningful connections between heterogeneous biomedical concepts is critically important [7]. Ontology matching is a solution to such semantic heterogeneity problem by determining the correspondences between concepts in different biomedical ontologies.

Because constructing alignments manually is time-consuming and labor-intensive, especially for large ontologies with thousands of concepts, some matching methods have been proposed to automatically generate ontology mappings [8]. These methods can be divided into 3 categories: terminological, structural, and external. Terminological methods are string based and designed to match names or name descriptions of ontology elements. Structural methods exploiting various types of ontology information, such as elements names, comments, and structural hierarchies, are proposed to compensate for the morphological differences between identical elements [8-14]. External methods obtain semantic mappings between syntactically dissimilar ontologies using auxiliary sources, such as taxonomies, dictionaries, and thesauri [15-18]. With the advancement of deep learning, there also exist some studies (eg, DeepAlignment [19], SCBOW + DAE(O) [20]) that try to discover alignments with representation learning based on deep learning. In the biomedical domain, some ontology matching methods based on deep learning have demonstrated the potential to facilitate the interoperability between ontologies [20-22].

Meanwhile, among the various matching techniques, to the best of our knowledge, there are surprisingly few systematic studies about the extraction and combination of matching clues and methods. As achieving satisfactory ontology alignments with a single technique is difficult, a composite approach is more efficient where different criteria or properties are considered within a single dimension. A composite approach, by contrast, that incorporates the results of some individual matchers may be simple or hybrid. This allows for high flexibility, as there is the potential for selecting the match algorithms to be executed based on the biomedical matching tasks. Moreover, there are different possibilities for combining the individual matching results. This paper attempts to empirically investigate and analyze the effectiveness of matching clues and the hybrid matching approaches.

Additionally, the inherent heterogeneity and large scale of biomedical ontologies have made discovering alignments a computationally intensive task. The divide and conquer approach [23,24] and ontology modularization [25] techniques have been proposed to decompose a large matching problem into some smaller submatching tasks. It does, however, have 2 limitations. First, most existing ontology partitioning approaches are unable to control the size of modules [23]. Consequently, many unproportionate modules (either too small or too large), which are inappropriate for matching, may be generated. Second, partitioning ontologies into modules may lead to the loss of valuable semantic information regarding the boundary elements. As a consequence, the quality of ontology matching may be impacted. Therefore, we extend *Reduction Anchors* [26], our previous method for dealing with large-scale ontology matching, to improve the performance of matching large-scale biomedical ontologies. Extended positive reduction anchors utilize the concept hierarchy to predict the ignorable similarity calculations, while the negative reduction anchors obtain the ignorable similarity calculations based on the locality of matching. The proposed method has 2 advantages over previous studies. First, it does not need to partition ontologies while maintaining the high performance as the divide and conquer approaches. Second, it is indeed a general large ontology matching framework, in which most existing matching techniques could be used.

Our main contributions in this paper are as follows:

- We provide several kinds of individual matchers with the utilization of different matching atomic clues. In order to investigate the effect of different clues in different dimensions, various combination strategies are studied to match biomedical ontologies.
- We represent multiple matchers in 4 dimensions: terminology, structure, external knowledge, and representation learning. To systematically examine and compare the effectiveness of different hybrid matchers, we design various matching strategies and combine the individual matchers for biomedical ontology matching tasks.
- We propose the extended reduction anchors-based approach for matching large-scale biomedical ontologies. It not only solves the scalability problem, but also achieves good performance with a significant reduction of execution time. Our approach achieves F1 measures of 0.925, 0.820, and 0.523 on Anatomy, FMA-NCI, and FMA-SNOMED, respectively, and reduces the matching time by nearly one-tenth. The high coverage (minimal information loss) achieved, combined with the reduction of the search space and the decreasing computation times, indicates that the extended reduction anchors are efficient.

### Related Work

In recent years, ontology matching has become a popular research field. Euzenat and Shvaiko [8] present a comprehensive overview of matching approaches and categorize techniques as terminological, structural, external, and representation learning dimensions [8]. We will focus on discussing related work on ontology matching of the biomedical domain.

### Biomedical Ontology Matching

According to the features used in ontology matching, matching approaches can be classified into 4 categories: terminology-based approach, structure-based approach, external knowledge–based approach, and representation learning–based approach.

#### Terminology-Based Approach

In the biomedical domain, discovering alignments relying on dictionaries and similarities of terms and labels is a typical ontology matching approach, which is still widely used [8]. In some matching systems such as ASMOV [15], SAMBO [27], Falcon [28], and AgreementMakerLight [16], the terminological matcher is exploited as a basic matching method. However, the terminology-based approach often provides good precision but a low recall because it is difficult to deal with variations in the form of terms or labels (eg, equivalence between *hindlimb bone* and *bone of the lower extremity*).

#### Structure-Based Approach

According to the intuition that elements of 2 distinct ontologies are similar when their adjacent elements are similar, structure-based matchers utilize property attributes and taxonomy hierarchy structure [29]. CroMatcher [30] focuses on the aggregation of distinct matchers in structural level: super-element matcher, subelement matcher, domain matcher, and range matcher. Similarity flooding [29] presents a structural algorithm based on fixpoint computation and propagation of similarities along with the property relationships between elements that are usable across different scenarios, including biomedical applications. Falcon-AO [28] uses a linguistic matcher combined with a technique that represents the structure of the ontologies to be matched as a bipartite graph. Besides, the similarities between domain elements and between statements in ontologies are computed by recursively propagating similarities in the bipartite graphs. FCA-Map [31] constructs relation-based formal context to describe the biomedical elements in taxonomic, partonomic, and disjoint relationships with the anchors, and then uses the context to validate the initial lexical mappings. LogMap [17] combines the structural indexation to represent the extended class hierarchy. Contexts for the same anchor are expanded by using the class hierarchies of the input biomedical ontologies to discover new mappings.

#### External Knowledge–Based Approach

Matching strategies based on external knowledge provide additional lexical or structural information, allowing for the obtaining of new alignments. Biomedical ontology matching systems explore potential resources or auxiliary knowledge, such as upper-level ontology, WordNet [32], UMLS [33], and BioPortal [34], to find synonyms, spelling variants, and annotations for the concepts to be matched. Systems such as LogMap-Bio [35] and AgreementMakerLight [16] exploit a set of ontologies as background knowledge to generate equivalent mappings. In addition to the anchoring mappings related to the same background ontology, Annane et al [36] utilize alignments produced by matching intermediate ontology between each other. Faria et al [37] present a novel approach based on building the specific mapping graph as background knowledge and take into account the limitation of the selection and the combination of heterogeneous existing mappings stored in a biomedical repository. It allows getting high-quality alignments between biomedical ontologies without using complex lexical and structural measures.

#### Representation Learning–Based Approach

Representation learning is so far rare in ontology matching, particularly in biomedical ontologies. There are a few approaches exploring unsupervised representation learning techniques to capture the interactions among element’s descriptions within biomedical ontologies. Zhang et al [38] investigated the use of representation learning for ontology matching and presented a hybrid method to incorporate word embeddings into the computation of semantic similarities among elements. Wang et al [39] proposed a neural architecture for biomedical ontology matching called OntoEmma [39]. It encodes a variety of descriptions, and derives large amounts of labeled data from biomedical thesaurus for training the model. Considering the problem of distinguishing semantic similarity and descriptive association on rare phrases, Kolyvakis et al [20] proposed a representation learning method: SCBOW+DAE(O) [20]. This approach is a representation framework based on terminological embeddings, in which the refinement of pretrained word vectors is introduced and learned by the domain knowledge encoded in ontologies and semantic lexicons. However, there still exist the limitations of the sparsity problem of structural relations and heavy dependence on pretraining. MultiOM [22] models the matching process by embedding techniques from multiple views and then optimizes the vector of concepts through a novel proposed negative sampling skill designed for structural relations in biomedical ontology.

Generally, multiple kinds of ontological clues are available, but matching biomedical ontologies based on a single category is constrained to achieve ideal performance. Consequently, most current matching systems, such as [15-17], focus on the hybrid and composite combination of various clues and matchers. Most composite methods, however, are confined to the customized combination of different matching clues and algorithms. By contrast, we attempt to study and evaluate multiple individual matchers with different combinations of matching clues and methods using different strategies. In addition, a systematic comparison of different matching clues and their integrations based on well-defined description clues does not exist so far.

### Large-Scale Biomedical Ontology Matching

Many matching systems cannot work well when dealing with large matching problems. These systems perform an all-against-all comparison between concepts of the input ontologies, which requires quadratic complexity n^2^ of similarity computing. To avoid the Cartesian product of the concept pairs of the source and the target ontologies, reduction of search space is indispensable.

Ontology modularization [40-42] aims to extract modules from a large and complex ontology, which is self-contained and logically consistent and can speed up the reasoning process and optimize memory utilization. Modular ontology is a popular way to partition large ontologies. However, existing modular ontology methods focus on the correctness and completeness of logics but cannot control the size of modules [23,27,43,44], that is, they would generate too large or too small modules. Algergawy et al [45] developed a seeding-based partitioning approach (OAPT) and introduced an information theoretic model selection method. It makes use of Bayesian information criterion (BIC) to determine the optimal number of modules that should be generated. However, the size of partitioned module remains uncertain.

Malasco [46] and Falcon-AO [28] are based on the divide and conquer approach that partitions a large ontology into a set of small clusters or blocks. Malasco employs 3 ontology partitioning algorithms: naive algorithm based on Resource Description Framework (RDF) sentences, structure-based algorithm [47], and ontology modularity based on ε-connection [40] for matching. Falcon-AO utilizes structural clustering to initially partition the ontologies into relatively small and disjoint blocks.

Although the modularization and divide and conquer approaches are effective to reduce the execution time, they still suffer from the contradiction between semantic completeness and information loss. After partitioning, ontology elements near boundaries of modules may lose some essential semantics, lowering the quality of alignments [26]. To overcome this problem, we introduce 2 kinds of reduction anchors to mitigate the impact of boundary loss, and simultaneously are able to reduce the number of entity pairs for which the similarity should be calculated during ontology matching.

## Methods

### Problem Formulation

An ontology is composed of triples like <*s*, *p*, *o*>, where *s*, *p*, and *o* stand for the subject, predicate, and object, respectively. There are 3 kinds of ontology resources: uniform resource identifier (URI) resources, literals, and blank nodes. In a triple, the subject can be URIs resources or blank nodes but not literals, and the predicate must be URI resources.

### Ontology

Let *O* be the RDFS (RDF Schema) or OWL (Ontology Web Language) ontology represented by a set of RDF triples *T*. The RDF triple *t*(*t*·*T*) denotes a statement in the form of *<subject, predicate, object>*. Any node in an RDF triple may be a URI with an optional local name, a literal, or a blank node. An ontology can be represented as *O* = (*C*, *R*, *I*), where *C, R,* and *I* denote sets of atomic concepts, relations (also named properties), and individuals, respectively. For simplicity, the set of concepts and properties is indicated by *E.*

We follow the work in [8] and give a formal definition for the ontology matching problem.

### Ontology Matching

The matching between 2 ontologies *O*_1_ and *O*_2_ is *M* = {*m_k_*|*m_k_* = <*e_i_*, *e_j_*, *r*, *s*>}, where *M* is an alignment; *m_k_* denotes a correspondence with a tuple <*e_i_*, *e_j_*, *r*, *s*>; *e_i_* and *e_j_* represent the expressions which are composed of elements from *O*_1_ and *O*_2_, respectively; *r* is the semantic relation between *e_i_* and *e_j_*; *r* could be equivalence (=), generic/specific (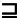/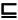), disjoint (⊥), and overlap (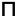), etc.; and *s* is the confidence about an alignment and typically in the [0,1] range. Therefore, an alignment *M* is a set of correspondences *m_k_*.

**Figure 1:**
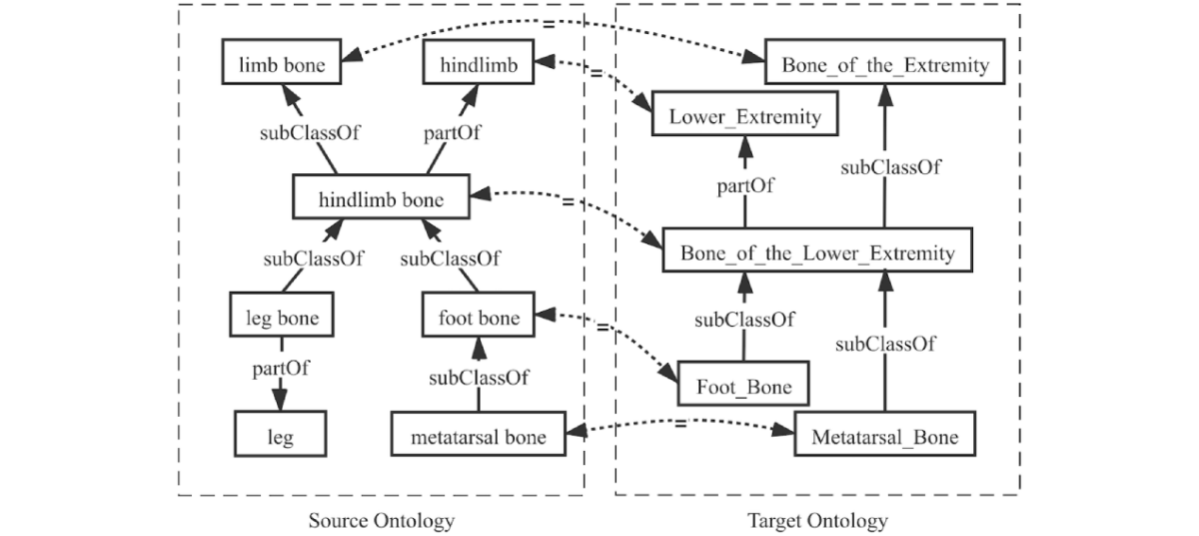
An example of biomedical ontology matching.

Figure 1 shows an example of alignments between a mouse anatomy ontology and the NCI Thesaurus. *<hindlimb bone, Bone_of_Lower_Extremity,=,0.7>* and *<limb bone, Bone_of_the_Extremity,=, 0.8>* are equivalent correspondences. In this paper, we only focus on identifying one-to-one equivalence correspondences between 2 concepts belonging to different ontologies.

### Biomedical Ontology Matching Framework

**Figure 2:**
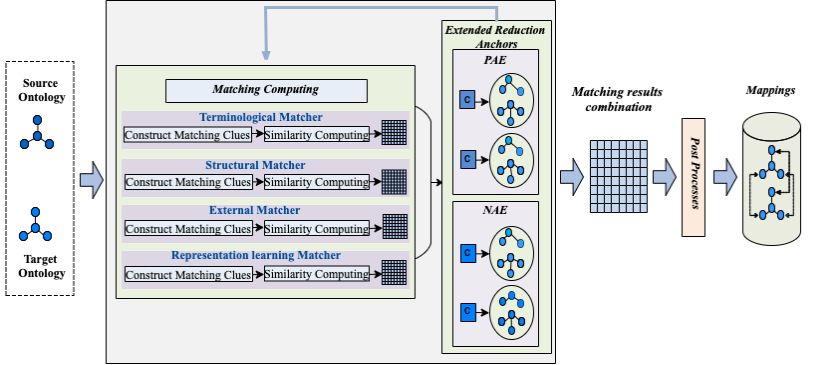
Overview of biomedical ontology matching. PAE: extended positive anchors; NAE: extended negative anchors.

Figure 2 depicts an overview of our biomedical ontology matching framework, which includes 3 steps: (1) constructing matching clues in different dimensions: terminology, structure, external knowledge, representation learning, and building different matchers based on the extracted clues to calculate the similarities between elements; (2) constructing and updating the extended reduction anchor set iteratively through the similarity results of each matching computation and skipping the ignorable computations based on the anchors set; and (3) combining similarity matrices of different matchers assigned with different weights to obtain the alignments. For each element in input ontologies, we first create matching clues in the form of virtual documents based on the re-defined dimensions, and then single matchers are built based on the extracted clues in each dimension. Then, the similarity matrix is measured by the similarity between corresponding documents of elements. According to the similarity of each pair of elements, extended reduction anchors sets are updated and optimized continuously, which are helpful to skip meaningless similarity computations and minimize time complexity as well as search space. After obtaining similarity matrices, predefined weights are assigned to each single matcher and the matching results are combined based on re-defined superiority. Finally, the alignments are obtained through filtering processing with a given threshold.

### Matching Clues

Generally, biomedical ontology generalizes and summarizes the categories of elements in the biomedical domain. Beyond names, ontology is concerned with the principled definition of biological classes and the relations between them. Apart from the knowledge contained in the ontologies, some external resources and semantic models can be exploited to enrich the element, potentially improving ontology matching efficiency. In this section, we describe the atomic clues available for ontology matching followed by the composite clues.

#### Atomic Clues

##### Overview

The atomic clues in ontology matching are given in Table 1, and are divided into 4 types: terminological clues, structural clues, external clues, and representation learning clues. For the nodes declared in an OWL/RDF ontology, we construct virtual documents to define their clues.

**Table 1:** Atomic clues for ontology matching.

Clues and their sources			Description
**Terminological**			
	Local name	Words in the local name of *e*	
	Label	Words in the rdfs:label of *e*	
	Comment	Words in the rdfs:comment of *e*	
	Synonym	Words in the synonym statements such as {*owl: sameAs*} and {*rdfs: seeAlso*}	
**Structural**			
	Property	Property attributes of concepts: property name, domain, range, and constraints	
	Hierarchy	Hierarchical context of concepts or properties, containing ancestors, descendants, siblings, and disjoint elements	
**External**			
	General dictionary	Retrieval of alternative labels and synonyms from general dictionaries such as WordNet, BabelNet	
	Lexicon	Cross-searching synonyms as well as cross-references from specific-domain thesauri	
**Representation learning**			
	General model	The embeddings of elements via general pretrained language models such as Word2Vec and BERT^a^	
	Specialized model	The embeddings via domain-specific pre-trained models such as BioBERT	

^a^BERT: Bidirectional Encoder Representations from Transformers.

##### Terminological Clues

The terminological clues are generally the direct and representative information that distinguishes between elements. As shown in Figure 1, the concepts *foot bone* and *metatarsal bone* in source ontology are equivalent to *Foot_Bone* and *Metatarsal_Bone* in target ontology, respectively. It illustrates that terms of elements are important clues for ontology matching. The terminological clues include the words in local names, comments, labels, and synonyms in triples with the predicates: rdfs:seeAlso, owl:sameAs, owl:hasExactSynonym, and owl:hasRelatedSynonym.

##### Structural Clues

While lacking sufficient and consistent linguistic information about the elements, ontology structure is a piece of useful information for finding alignments. In Figure 1, for *hindlimb bone* of source ontology and *Bone_of_Lower_Extremity* of target ontology, it is difficult to discover the mapping through the terminological representations. But they have similar neighbors, *foot bone* and *Foot_Bone*, based on which we can infer that the 2 concepts would be similar. For the structural clues, they could be divided into property clues and hierarchy clues.

The property clues contain the properties attributes of concepts. The properties are represented with name, domain, and range. Some constraints might be associated with these properties, for instance, the notion of functional property.

The hierarchy clues are the context of the corresponding elements, which are reflected by ancestors, descendants, siblings, and disjoint nodes. The direct children reflect its basic structure, while the leaves reflect its semantic context. (1) The ancestor context of a node *n_i_* could be the descriptions of upper nodes that directly link to *n_i_* or parent nodes within a given hierarchical depth. For a blank node, likewise, we obtain the ancestor context through recursively forward traversing until the occurrence of the nonblank nodes. (2) The descendant context of a node *n_i_* could be the set of basic and extensional descriptions of its nearest subelements or leave nodes of the subtrees of the node. Because the context of a blank node is an empty set, we could recursively obtain the context from the set of leaf nodes of subtrees rooted at node *n_i_*. (3) The sibling context of a node *n_i_* is defined as a set of linguistic descriptions of nodes in the same hierarchy with *n_i_*, and these nodes share the same parent with node *n_i_*. (4) The disjoint context of *n_i_* is defined as the collection of linguistic descriptions of nodes that are disjoint with *n_i_*.

##### External Clues

To compensate for the lack of structure and lexical information, some auxiliary knowledge and external representations are used to extract further alignments.

We retrieve alternative labels for elements to be mapped from general dictionaries (ie, WordNet). In addition, considering the specialization of biomedical ontology matching, domain-specific ontologies such as UBERON [48] and UMLS [33] are employed as auxiliary information. These ontologies are exploited to extract the cross-references and alternative synonyms, which are available to identify additional anchors.

##### Representation Learning Clues

Apart from the features of terminological, structural, and external resources, representation learning also has the potential to bring more semantics to biomedical ontology matching.

Word embedding can represent the implicit semantics behind elements. The general pretrained models, for example, Bidirectional Encoder Representations from Transformers (BERT) [49], trained on the large text corpus could be used to encode the element and then compute the alignments. Domain-specific language representation models are more preferable to obtain the embeddings of elements within biomedical ontologies. BioBERT [50], a domain-specific language representation model pretrained on large-scale biomedical corpora, might perform better in capturing the semantic of biomedical classes than the general models. Furthermore, fine-tuning BioBERT with synonym marginalization algorithm presented in [51] is expected to improve the quality of element representation.

#### Composite Clues

The composite clues are the combinations of atomic clues with different weights. The composite clues of the element *e* are constructed as follows:

Clue(*e*)= *α*_1_ * Term(*e*) + *α*_2_ * *Struc*(*e*) + *α*_3_ * *Ext*(*e*) + *α*_4_ * *Rps*(*e*)

where *α*_1,_ *α*_2,_ *α*_3,_ and *α*_4_ are weights in [0,1], and *Term*(*e*), *Struct*(*e*), *Ext*(*e*), and *Rps*(*e*) denote the terminological clues, structural clues, external clues, and representation learning clues of *e*, respectively.

### Matching Process

In this section, we describe in detail the overall biomedical ontology matching process.

#### Name Matcher

Element names represent an important information for accessing similarities. However, in some ontologies, the local names of elements are represented in the form of ID, such as *NCI_C12269* in NCI Thesaurus and *MA_0000216* in MA ontology, which is meaningless. Consequently, we first simply obtain the mapping results through comparing the label sets of the pairs of elements. The normalized edit distance similarity metric is applied to compute linguistic similarities between label sets:

SIM_name_ = DNE(*s,t*) = [DE(*s,t*)]/[DE(*s,t*) + SE(*s,t*)]

SE(*s,t*) = [(|*s*| + |*t*| – DE(*s*,*t*))]/2

where DE(*s,t*) denotes the edit distance between string *s* and *t*, and SE(*s*,*t*) denotes the edit similarity between *s* and *t*. The normalized edit distance similarity is denoted as DNE(*s,t*), and the function |*x*| denotes the length of *x*. After that, mapping results are generated through a given threshold filtering and similarity ranking.

#### Terminology Matcher

Biomedical ontologies are characterized by terminological components in the form of names and various types of synonyms along with comments. We combine the labels with corresponding extensional clues to get the similarity between 2 elements. We chose term frequency-inverse document frequency (TF-IDF) to measure the similarity between the terminological documents of element *e_s_* from source ontology and element *e_t_* from target ontology:

SIM(*x*, *y*) = TF*IDF

TF = *w*/*W*

IDF = 1/2*(1+log_2_[N/*n*])

SIM_term_(*e_s_*,*e_t_*) = SIM(*Term*[*e_s_*], *Term*[*e_t_*])

For each description document, *w* denotes the refined word occurrence; and *W* denotes the total refined occurrence among all the words in a specific document. And finally, we select the matching pairs with maximum similarity values.

#### Structure Matcher

The structural clues, including hierarchies (subclass, superclass, sibling class, disjoint class) and property attributes (domain, range), allow more possible candidate mappings to be discovered. The structural similarity of the element relies on the similarity of context descriptions. The extracted context set of each node may contain the terminological clues of direct neighbors, of adjacent nodes within local graph, that is, extracted semantic subgraph [52], or adjacent nodes in global graph. The similarity value of structural clues between each pair of nodes is initially measured by TF-IDF similarity between the structural documents:

SIM*_struc_*(*e_s_*,*e_t_*)=SIM(*Struct*[*e_s_*],*Struct*[*e_t_*])

#### External Matcher

Two kinds of external resources are used to further promote the matching. From the general dictionary, synonyms are retrieved by the name of element. Meanwhile, from the domain-specific repository, equivalent classes are obtained based on terms of elements, and then discovering the synonyms of the discovered classes. In addition, cross-references are extracted based on the property *DbXref*. The extracted synonyms are viewed as the extensional comments of corresponding elements, and cross-references are used as the reference alignments. TF-IDF is used to determine the degree of each pair of collections of thesaurus information, respectively:

SIM*_ext_*(*e_s_*,*e_t_*)=SIM(*Ext*[*e_s_*],*Ext*[*e_t_*])

#### Representation Learning Matcher

For the chosen pretrained language models, either BERT or BioBERT, and refined models, the input is a mention–synonym pair, and the outputs of the last hidden layer are concatenated to represent the mention. After getting the embeddings of ontological terms, we then use the cosine distance over the pairs of embedding representations as the similarity score:

SIM*_rps_*(*e_s_*,*e_t_*)=SIM(*Rps*[*e_s_*],*Rps*[*e_t_*])

#### Hybrid Matcher

To obtain more accurate similarity values, the hybrid matchers are constructed through a fixed combination of simple matchers and other hybrid matchers. A straightforward strategy is summing up the values of all match results and getting the averages to denote the similarity between each pair of elements. However, it may introduce lots of wrong mappings due to the neglect of differences between matchers. Therefore, we combine the similarity matrices by assigning varying weights to reflect their importance.

### Matching Large Biomedical Ontologies

#### P-Anchors and N-Anchors

To deal with the scalability problem of large biomedical ontology matching, this paper extends the matching method based on reduction anchors with related nodes. The reduction anchors–based approach [26] is desirable for matching large ontologies. It utilizes positive reduction anchor (P-Anchors), based on the coherence of structural hierarchy of ontology alignment, and negative reduction anchor (N-Anchors), based on the locality characteristic of matching, to reduce undesirable comparisons. However, because matching based on P-Anchors is highly dependent on the hierarchical depth of ontology while usually the average depth of biomedical ontologies is typically limited, the matching cannot achieve the ideal high performance. Therefore, we extend reduction anchors with the related nodes to refine the matching. In this section, we first introduce the definition of our improved extended reduction anchors and then present the matching method based on extended reduction anchors.

#### Reduction Anchors

##### Extended Positive Reduction Anchor (P-AnchorE)

Given a concept *a_i_* in ontology *O*_1_ with equivalent concept set *a_i_*_1_, *a_i_*_2_, …, *a_im_*, let the similarities between *a_i_* and concepts *b*_1_, *b*_2_, …, *b_n_* in ontology *O*_1_ be *S_i_*_1_, *S_i_*_2_, …, *S_in_*, respectively. If *S_ij_* is larger than the predefined threshold *ptValue*, the concept pair (*a_i_*, *b_j_*) is a positive reduction anchor, and all positive reduction anchors about *a_i_* are denoted by PA(*a_i_*) = {*b_j_*|*S*_ij_ > ptValue}. Then, the extended positive reduction anchor of *a_i_* is 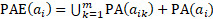.

##### Extended Negative Reduction Anchor (N-AnchorE)

Given a concept *a_i_* in ontology *O*_1_ with equivalent concept set *a_i_*_1_, *a_i_*_2_, …, *a_im_*, let the similarity values between *a_i_* and concepts *b*_1_, *b*_2_, …, *b_n_* in ontology *O*_2_ be *S_i_*_1_, *S_i_*_2_, …, *S_in_*, respectively. If *S_ij_* is smaller than the predefined threshold *ntValue*, the concept pair (*a_i_*, *b_j_*) is a negative reduction anchor, and all negative reduction anchors about *a_i_* are denoted by NA(*a_i_*) = {*b_j_*|*S_ij_* < *ntValue*}. Then, the extended negative reduction anchors of *a_i_* are as follows:



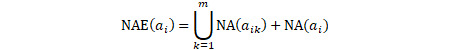



#### LOM-PE: Large Ontology Matching Algorithm Based on P-AnchorsE

Let PSE(*a_i_*), the extended positive reduction set of *a_i_*, be all the ignorable similarity calculations predicted by PAE(*a_i_*). If |PAE(*a_i_*)| > 0, we select the top-*k* P-AnchorsE with maximum similarities. Let PS(*a_i_*) be the initial positive reduction set about a P-AnchorE (*a_i_*, *b_j_*), which is calculated as follows:



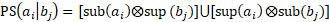



Meanwhile, the reduction computation can be propagated to the concepts that are highly similar to sub(*a_i_*). Therefore, sub(*a_i_*) can be extended as follows:



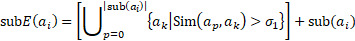



Plus, sup(*a_i_*), sub(*b_j_*), and sup(*b_j_*) can be calculated analogously. Then the extended reduction set of PSE(*a_i_*|*b_j_*) is:



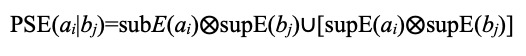



If PSE(*a_i_*) = {*b*_1_, *b*_2_, …, *b_k_*}, the corresponding extended reduction set can be calculated as follows:



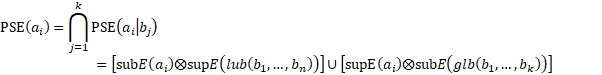



where *lub*() and *glb*() are the functions to obtain the least upper bound and greatest lower bound, respectively. The formula above indicates that smaller *top-k* will generate larger PSE(*a_i_*). In our implementation, *top-k* is assigned a value from 1 to 4. The total positive reduction set during matching is:



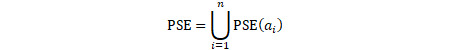



Multimedia Appendix 1 presents a large ontology matching algorithm based on P-AnchorsE (LOM-PE). Here, LOMPE-Algorithm() is the main function, ComputerSim() matches elements on the hierarchy path recursively, and GetPAnchorsE() obtains top-*k* P-AnchorsE.

The time complexity of the LOM-PE algorithm is analyzed as follows: Given 2 matched ontologies, if all concepts are on a hierarchy path, the matching process can generate *n*(*n*–2) size valid positive reduction set, and it just needs 2*n* similarity calculations, that is, the algorithm has the best time complexity *O*(2*n*). However, such an ideal case almost does not exist in the real world. Suppose there are *m* hierarchy paths, then the average depth of the ontology is 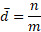. Consequently, we can derive the time complexity of Multimedia Appendix 1 as follows:



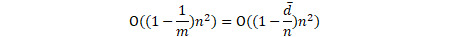



#### LOM-NE: Large Ontology Matching Algorithm Based on N-AnchorsE

The set of all ignorable similarity calculations predicted by N-AnchorsE is called the extended negative reduction set. Let *Nb*(*a_i_*) = {*a_x_*|*d*(*a_x_*,*a_i_*) <= *nScale*} be the neighbors with *nScale* distance to *a_i_*. Therefore, the initial negative reduction set generated by *a_i_* is:



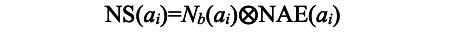



According to the formula, NAE(*a_i_*) will be propagated to neighbors of *a_i_*. And there are 3 constraints being introduced to reduce the risk of low credible negative reduction set: (1) all N-AnchorsE must be obtained in similarity calculating; (2) all N-AnchorsE of *a_i_* can only be propagated to the neighbors in the semantic subgraph of *a_i_*; and (3) all N-AnchorsE of *a_i_* can be propagated only if the description document of *a_i_* contains more than *t* items.

Similar to LOM-PE, the reduction computation can also be propagated to the concepts that are highly similar with *Nb*(*a_i_*). Therefore, the extended neighbors set is as follows:



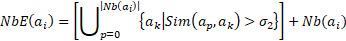



Then, the final extended negative reduction set can be denoted as:



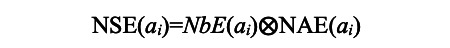



where the extended set NSE() should also comply with above 3 constraints.

Multimedia Appendix 2 presents a large ontology matching algorithm based on N-AnchorsE (LOM-NE). All concepts are sorted by their degrees (line 2). If a similarity *s* is smaller than *ntValue* and satisfies 3 constraints (line 9), an N-AnchorE (line 10) is used to get the extended negative reduction set (line 12). After refining the extended negative reduction set, we obtain the valid extended negative reduction set (line 13). The time complexity of the algorithm is *O*([1–*wλ*]*n*^2^), where *w* is the average degree and *λ* is determined by *ntValue* and constraints. The bigger *w* and *λ* are, the higher performance the algorithm has.

#### LOM-Hybrid: Hybrid Large Ontology Matching Algorithm

We use a hybrid algorithm, called LOM-Hybrid, to combine the LOM-PE and LOM-NE algorithms to obtain as large a valid reduction set as possible. It can be a benefit for the LOM-PE algorithm if the LOM-NE algorithm that calculates the elements with large degree is implemented first: (1) Because the average depth of a real ontology is often small, while LOM-PE relies on the depth of concept hierarchy, the LOM-PE might not have an ideal performance. (2) The elements with large degree, most of which are located in the middle of hierarchy, would be calculated first by LOM-NE, and it can benefit the LOM-PE. Therefore, the LOM-Hybrid algorithm is mainly based on the framework of the LOM-NE algorithm, in which the LOM-PE algorithm is embedded. LOM-Hybrid can generate the valid positive reduction set and negative reduction set. Theoretically, the time complexity of LOM-Hybrid is between the complexity of LOM-NE and the complexity of LOM-PE. Indeed, it is very close to LOM-NE. However, in the real-world cases, the actual time complexity is indeed close to that of LOM-NE.

## Results

### Overview

We performed a comprehensive evaluation of the match processing strategies on real-word ontologies. The main goal is to investigate the impact of different combination strategies, that is, selection and aggregation of clues, on match quality, and to compare the effectiveness of different matchers, that is, single matcher and different combinations of individual matchers. We used Java to implement our approaches and conduct the experiments on a computer with an Intel Xeon 4110 CPU and 64-GB memory.

### Data Set

Our experiments are conducted on 4 ontologies that appear in the Ontology Alignment Evaluation Initiative (OAEI). Two of them (the Adult Mouse Anatomy Ontology and the Foundational Model of Anatomy) are pure anatomical ontologies, while the other 2 (SNOMED-CT and NCI Thesaurus) are broader biomedical ontologies.

Adult Mouse Anatomy is a structured dictionary that provides standardized nomenclature for anatomical terms in the postnatal mouse and organizes anatomical structures for the postnatal mouse spatially and functionally [53].

Foundational Model of Anatomy (FMA) is an evolving computer-based knowledge source for biomedical informatics. The FMA is a domain ontology of the concepts and relationships that pertain to the structural organization of the human body [2].

NCI Thesaurus (NCI) provides reference terminology for many NCIs and other systems. It covers vocabulary for clinical care, translational and basic research, public information, and administrative activities [1].

SNOMED-CT is a systematically organized computer-processable collection of medical terms providing codes, terms, synonyms, and definitions used in clinical documentation and reporting [3].

The detailed statistics of each ontology matching task are presented in Table 2. For Anatomy, there are 2737 concepts in source ontology and 3298 concepts in target ontology, simultaneously including many labels and synonyms but only the *PART_OF* property with both ontologies. FMA-NCI task selects a small part of FMA and NCI ontology, with 3696 concepts from FMA and 6488 concepts from NCI, and FMA-SNOMED also selects a fragment of these ontologies with tens of thousands of concepts, 10,157 concepts in the source ontology FMA and 13,412 concepts in the target ontology SNOMED. For FMA-NCI and FMA-SNOMED, there exists no synonym within the ontologies but some properties to define the relations between entities, 24 properties for FMA, 63 properties for NCI, and 18 properties for SNOMED. For each concept, there are almost several aliases (labels) that are important for the alignments of heterogeneous ontologies. The evaluation of tasks is summarized through the MELT (Matching EvaLuation Toolkit) framework supported in OAEI. Actually, the alignments of tasks FMA-NCI and FMA-SNOMED are conducted on a small fragment of the aforementioned ontologies.

**Table 2:** Summary statistics of the biomedical ontology matching tasks.

Task and ontology			#Concepts		#Labels		#Synonyms		#Properties		#Triples
**Anatomy**											
	MA	2737		3084		344		2		15,958	
	NCI^a^	3298		9403		5246		1		35,354	
**FMA-NCI**											
	FMA^b^	3696		9142		0		24		16,919	
	NCI	6488		17,109		0		63		64,857	
**FMA-SNOMED**											
	FMA	10,157		26,989		0		24		47,730	
	SNOMED	13,412		13,431		0		18		110,029	

^a^NCI: National Cancer Institute.

^b^FMA: Foundation Model of Anatomy.

### Measures

In order to measure the performance of the matching system, we selected precision, recall, and F-measure adapted for ontology matching evaluation.

We compare the mapping *M*, which consists of all those correspondences generated by our system, against reference mapping *R* to compute precision *p*, recall *r*, and F1-measure *F*. The standard measures for evaluating mappings are denoted as follows:

*p*(M,R)=(|M∩R|)/M

*r*(M,R)=(|M∩R|)/R

*F*(M,R)=[2·*p*(M,R)·*r*(M,R)]/[*p*(M,R) + *r*(M,R)]

### Experiment Settings

We define several hybrid matchers in different combinations of atomic clues and matching dimensions. The details of designed matchers are listed in Table 3. For the clues in this table, *syn* means the sets of synonyms of concepts, *prop* is the abbreviations of property, *dh* denotes direct hierarchy utilizing the nearest neighbors, *lh* is the local hierarchy using structural clues within corresponding semantic subgraphs, and *gh* represents the global hierarchy that uses global structure based on transitive rules. In addition, *WN* denotes the general dictionary WordNet selected in our notion, and *U_dic_* denotes the domain-specific dictionaries, UBERON and UMLS, in our experiments. For the representation learning clues, we choose BERT as the general model; and BioBERT and fine-tuned BioBERT denoted as fBio as the specialized representation models.

There are 3 terminological matchers, 5 structural matchers, 2 external matchers, and 3 representation learning matchers. The comment is absent in the data sets, so we eliminate it in atomic clues. In the ontologies of the Anatomy task, the local names of elements are in the form of ID, and for the largebio tasks, the names are wholly contained in labels. Thus, we mainly focus on the comparison of labels instead of names. Owing to the crucial role of terminological clues in ontology matching, the terminological matcher is constructed as the basis of matchers in the other dimensions.

**Table 3:** Relations between clues and matchers.

Matcher			Clue																						
			Name		Label		syn		prop		dh		lh		gh		WN		U_dic_		BERT^a^		BioBERT		fBio
**Terminological**																									
	*M*_1_	✓																							
	*M*_2_			✓																					
	*M*_3_			✓		✓																			
**Structural**																									
	*M*_4_			✓		✓		✓																	
	*M*_5_			✓		✓				✓															
	*M*_6_			✓		✓		✓		✓															
	*M*_7_			✓		✓						✓													
	*M*_8_			✓		✓								✓											
**External**																									
	*M*_9_			✓		✓										✓									
	*M*_10_			✓		✓												✓							
**Representation learning**																									
	*M*_11_			✓		✓																			
	*M*_12_			✓		✓																✓			
	*M*_13_			✓		✓																		✓	

^a^BERT: Bidirectional Encoder Representations from Transformers.

### Research Questions

We attempted to investigate the following research questions to understand the influence of different aggregations of ontology clues, to compare the effectiveness of different matcher combinations, and to verify the practical usefulness of extended reduction anchors.

#### Research Question 1 (Influence of the Combination Strategies of Clues): How Do the Different Combination Strategies of the Clues Perform in Ontology Matching?

The purpose of research question 1 is to investigate how a combination strategy influences the matching performance. There are generally several kinds of available clues in a single dimension. For instance, in the point of structure, there are intra structure and extra structure. However, some part of them may have a negative effect on the matching results. The study of research question 1 could help discover the influence of key clues during matching.

#### Research Question 2 (Effectiveness of Matcher Combinations): How Effective Are the Combinations of Matchers Implemented in Ontology Matching?

The purpose of research question 2 is to investigate whether utilizing the different aggregations of matchers could promote the matching effect, and to explore which combinations could be useful to match ontology. The study of research question 2 aims to learn how the integration of matchers influences the matching results.

#### Research Question 3 (Scalability of Reduction Anchors): What Is the Performance of Extended Reduction Anchors While Matching Large Biomedical Ontologies?

The purpose of research question 3 is to verify the effectiveness of our reduction anchors. Because of the large scale of biomedical ontologies, the matching is time-consuming and acquires amounts of space. As a result, there is a strong need to eliminate meaningless computations to reduce time and space complexity. The study of research question 3 is dedicated to demonstrate the effectiveness of resolving the scalability problem brought by the reduction anchor–based approach.

### Results of Terminology-Based Matcher

In the initial phase, we examine the matcher with the direct linguistic description of concepts: name, labels, synonyms, and comments.

Table 4 shows the matching results by utilizing terminological clues in the ontologies. For the task of Anatomy, there is no matching to be obtained for the name being in ID format. It can be observed that labels act as a strong distinguishing feature for matching biomedical ontologies, and relying on the string similarity of labels can achieve a fundamental precision and F1 measure, particularly for Anatomy and FMA-NCI. Besides, integrating internal synonyms to get the name variants can further improve the performance of system. Although it slightly decreases the precision of alignments, it can increase the recall and F1 measure distinctly. For the tasks FMA-NCI and FMA-SNOMED, there is no change in the metrics owing to the absence of synonyms in the input ontologies. In addition, we can find that the terminological matchers result in a substantial difference in the matching performance of different tasks. The terminological matcher achieves precision of 0.9658 and 0.9001 on Anatomy and FMA-NCI, respectively. But the precision on FMA-SNOMED is 0.3549. The Anatomy ontologies mainly focus on the anatomical terms of adult mouse anatomy and human anatomy whose terminological names are highly similar. By contrast, for the tasks of FMA-NCI and FMA-SNOMED, the ontologies cover a variety of topics and name the objects in different criteria, which cause the difference between their phenotypic representations.

**Table 4:** Results of terminology-based matcher.^a^

Method	Anatomy				FMA^b^-NCI^c^				FMA-SNOMED		
	P (%)	R (%)	F1 (%)	P (%)		R (%)	F1 (%)	P (%)		R (%)	F1 (%)
*M*_1_	0	0	—	86.30		53.74	66.23	25.22		28.14	26.60
*M*_2_	**96.58**	68.87	80.40	**90.01**		**70.90**	**79.32**	**35.49**		**36.59**	**36.03**
*M*_3_	92.28	**74.14**	**82.22**	**90.01**		**70.90**	**79.32**	**35.49**		**36.59**	**36.03**

^a^Values in bold indicate best experimental results.

^b^FMA: Foundation Model of Anatomy.

^c^NCI: National Cancer Institute.

### Results of Structure-Based Matcher

There are 5 structure-based matchers which consider different structural clues of ontologies. First, we measured the effect of property and hierarchy, respectively, while only the nearest neighbors are considered in the hierarchy. Then, we evaluated the results using a combination of both. In addition, the transitive closure is taken into account to get the global structure with predefined decay coefficients. Furthermore, we assess the results of the structural matcher which has the local structure within the constructed semantic subgraph for each concept.

Table 5 shows the matching results of different combination strategies of structural atomic clues. Specifically, we use G to denote the F1 measure difference between structure-based matchers and the best terminology–based matcher. Overall, G demonstrates that the structure has a positive impact on matching performance. In the Anatomy task, the property information slightly degrades the mapping results, the reason is that there are only 2 properties, *UNDEFINED_is_a* and *UNDEFINED_part_of*, which bring valueless information. It is evident that the hierarchical structure has a positive effect, which improves the F1 score by about 4%. When we choose the entire hierarchies extended with transitive rules, the recall and F1 measure fall a little compared with the direct structure that combines the clues of direct nodes, for the reason that it would bring about some redundancy and much more noise by integrating too many hierarchical clues. Furthermore, the recall achieves an impactful enhancement while utilizing extracted semantic subgraphs to capture the real meaning of concepts, but is accompanied by a decline in precision compared with some other matchers. In the task of FMA-NCI, the local structural clues can result in about 2% improvement. However, there is hardly an obvious change when we combine other different hierarchical information. Because of the relatively obvious morphological difference between different ontologies, utilizing the local structure to discover more semantically related entities could result in better performance compared with direct structural clues. Because of the large size of FMA-SNOMED, which takes too much time to recursively retrieve global structure and construct the semantic subgraphs, the results of *M*_7_ and *M*_8_ are ignored in our experiments. However, utilizing all clues cannot guarantee the improvement of mappings. For instance, exploiting the property has a little positive effect than extending clues with only the direct linking nodes.

**Table 5:** Results of structure-based matcher.^a^

Method	Anatomy				FMA^b^-NCI^c^					FMA-SNOMED					
	P (%)	R (%)	F1 (%)	G (%)		P (%)	R (%)	F1 (%)	G (%)		P (%)	R (%)	F1 (%)	G (%)	
*M*_4_	90.66	74.14	81.56	–0.66		88.36	72.82	79.85	+0.53		37.21	41.59	39.27	+3.24	
*M*_5_	91.82	81.40	**86.30**	**+4.08**		88.96	72.48	79.88	+0.56		37.25	41.68	39.34	+3.31	
*M*_6_	**92.23**	80.61	86.03	+3.81		**89.62**	72.28	80.03	+0.71		**37.29**	**41.88**	**39.46**	**+3.45**	
*M*_7_	88.04	**84.56**	86.27	+4.05		88.25	**74.93**	**81.05**	**+1.73**		—^d^	—	—	—	
*M*_8_	91.93	80.41	85.78	+3.56		89.36	72.49	80.03	+0.71		—	—	—	—	

^a^Values in bold indicate best experimental results.

^b^FMA: Foundation Model of Anatomy.

^c^NCI: National Cancer Institute.

^d^Not available.

### Results of External-Based Matcher

This section studies the performance of external-based matchers utilizing general dictionaries (WordNet) and external domain-specific knowledge (UBERON and UMLS). The experimental results of the 2 methods *M*_9_ and *M*_10_ are presented in Table 6.

We obtain the precedent sense of names through WordNet to enrich the synonyms of ontology concepts. Because WordNet is difficult to get relevant synonyms unless the sense of the term is known a priori and that compound terms are strongly covered, it brings a negative influence on all the 3 tasks. Then, UBERON and UMLS, which are related to biomedical science, are selected to further enrich ontology descriptions. On the one hand, we acquire all the correlative synonyms and cross-search references about the input ontologies. On the other, a reverse synonym lexicon is constructed, which is initiated by the idea that there may be a lack of description *Syn*(*b*) = *a* while *Syn*(*a*) = *b* exists. It can be observed that the specialized lexicon produces an effective influence compared with the common repositories. For Anatomy, specialized lexicon brings an increase of 8.3%, while the common lexicon causes a 1.33% decrease in F1 score. However, the domain-specific lexical brings about a much less positive effect on mapping results of FMA-NCI with an increase of 1.33%, and an increase of 8.3% and 13.49% for Anatomy and FMA-SNOMED, respectively. The synonyms and cross-search references are extracted from the auxiliary knowledge with the terminological names directly. However, there are a few available auxiliary clues for the mapping of FMA and NCI, which accounts for the little rise of the FMA-NCI task.

**Table 6:** Results of external-based matcher.^a^

Method	Anatomy					FMA^b^-NCI^c^					FMA-SNOMED			
	P (%)	R (%)	F1 (%)	G (%)	P (%)		R (%)	F1 (%)	G (%)	P (%)		R (%)	F1 (%)	G (%)
*M*_9_	90.05	73.42	80.89	–1.33	79.50		**74.01**	76.66	–2.66	32.38		36.48	34.31	–1.72
*M*_10_	**92.67**	**88.46**	**90.52**	**+8.3**	**89.65**		73.47	**80.75**	**+1.33**	**46.78**		**52.60**	**49.52**	**+13.49**

^a^Values in bold indicate best experimental results.

^b^FMA: Foundation Model of Anatomy.

^c^NCI: National Cancer Institute.

### Results of Representation Learning–Based Matcher

We select 3 pretrained language models to study the influence of word embedding. Table 7 shows the comparison of different word embedding techniques applied to biomedical ontology matching. Surprisingly, although BERT is trained by amounts of general corpora, it still has resulted in a slight decline in the results. It seems that capturing implicit semantics in a specific domain could be more difficult than we imagined. BioBERT is a domain-specific language representation model pretrained on large-scale biomedical corpora. It shows a slightly better performance than BERT, indicating that incorporating domain-specific language representation can be valuable to ontology matching. The fine-tuned BioBERT is trained on the correspondence between concepts and synonyms within UBERON which is much more relevant to the tasks. The results show that the fine-tuned BioBERT is able to produce much more positive influence on capturing the semantics in ontologies. In fact, the cost of representation learning technique outweighs the benefit that it can bring to our mapping tracks. Especially for the FMA-NCI, it results in a negative effect on the mapping performance for the reason that the training corpora is less relevant to FMA-NCI. In addition, there are restrictive synonyms within the ontologies of FMA-NCI and FMA-SNOMED, which is insufficient for training of representative learning matchers.

**Table 7:** Results of representation learning–based matcher.^a^

Method	Anatomy					FMA^b^-NCI^c^					FMA-SNOMED			
	P (%)	R (%)	F1 (%)	G (%)	P (%)		R (%)	F1 (%)	G (%)	P (%)		R (%)	F1 (%)	G (%)
*M*_11_	89.55	74.68	81.44	–0.78	87.94		71.47	78.85	–0.47	33.54		37.66	35.47	–0.56
*M*_12_	90.21	**76.32**	82.69	+0.47	**88.19**		71.93	79.23	–0.09	**35.04**		37.08	36.03	+0.00
*M*_13_	**93.31**	74.39	**83.02**	**+0.8** **0**	86.84		**72.97**	**79.30**	**–0.02**	34.22		**38.63**	**36.29**	**+0.26**

^a^Values in bold indicate best experimental results.

^b^FMA: Foundation Model of Anatomy.

^c^NCI: National Cancer Institute.

### Results of Matcher Combinations

After evaluating the single matchers in different dimensions, we conducted a series of experiments to examine the effectiveness of different combination strategies. For each selection strategy, we choose the optimal parameter range, in which the best match result is to be expected.

Table 8 shows the performance of hybrid matchers combined with different matchers. There are 7 hybrid matchers used in our experiments, where *Term*, *Struc*, *Ext*, and *Rps* represent the terminological matcher, structural matcher, external matcher, and representation learning matcher, respectively. Different combinations have different influences on different tracks, and integrating some matchers may thus exert negative effect on matching. We can observe that the combination of all 4 matchers, that is, *Term* + *Struc* + *Ext* + *Rps*, can achieve the best performance for Anatomy, while incorporating *Term*, *Struc*, and *Ext* together leads to the best results for FMA-NCI and FMA-SNOMED. As the representation learning matcher is trained with the synonym marginalization algorithm while there are less synonym clues within the ontologies of FMA-NCI and FMA-SNOMED compared with Anatomy, the *Rps* may be less helpful than the other 3 matchers for FMA-NCI and FMA-SNOMED.

**Table 8:** Results of hybrid matcher.^a^

Method	Anatomy			FMA^b^-NCI^c^			FMA-SNOMED		
	P (%)	R (%)	F1 (%)	P (%)	R (%)	F1 (%)	P (%)	R (%)	F1 (%)
Term	92.28	74.14	82.22	90.01	70.90	79.32	35.49	36.59	36.03
Term + Struc	92.23	80.61	86.03	88.25	74.93	81.05	37.29	41.88	39.46
Term + Ext	92.67	88.46	90.52	89.65	73.47	80.75	46.78	*52.60*	49.52
Term + Rps	93.31	74.39	83.02	87.94	71.47	78.85	33.54	37.66	35.47
Term + Struc + Ext	93.78	90.44	92.08	*90.64*	*75.17*	*82.18*	*47.97*	52.21	*50.00*
Term + Ext + Rps	91.56	88.92	90.22	88.90	72.95	80.14	43.76	47.83	45.70
Term + Struc + Ext + Rps	*94.95*	*90.57*	*92.71*	90.04	74.47	81.52	47.52	52.01	49.66

^a^Italicized values indicate best experimental results.

^b^FMA: Foundation Model of Anatomy.

^c^NCI: National Cancer Institute.

### Performance Evaluation of Matchers

**Figure 3:**
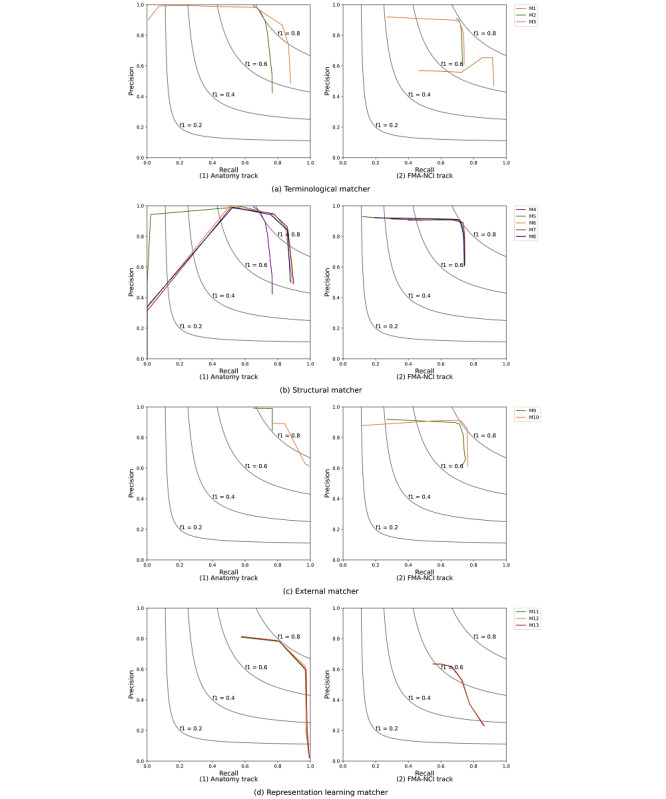
Comparison in PR space.

Here we present the metrics of precision and recall along with F1 measure of each matcher and analyze the correlation between them. In general, the higher the precision, the lower is the recall. However, there are also some exceptions when the similarity threshold is high enough. In Figure 3, the comparisons in the precision–recall space over 4 aspects of Anatomy and FMA-NCI track are depicted. From Figure 3, the terminological matcher that combines labels and synonyms could achieve the best results. For the structural matchers, most of them perform similarly and there is no obvious difference among them. For the FMA-NCI task, there is slightly a little difference in matching performance between most matchers. As for the external matchers, *M*_10_ utilizing domain-specific lexical has a significant difference with *M*_9_. However, for the matchers that are using the method based on representation learning, the precision–recall curve is obviously similar. This is because similar names may refer to different objects, while names in different morphologies may refer to the same object. Therefore, exploiting only the semantic representations remains hard to capture the true meaning for these elements of specific domain.

### Effectiveness of Extended Reduction Anchor

To examine the validity of the extended reduction anchors, we conduct comparison and evaluation through integrating extended reduction anchors with the best performing matchers. The results are indicated in Table 9.

We can learn from the table that our improved reduction anchors could benefit the time complexity during matching. According to the results shown in Table 9, the reduction anchors are more comparative while the ontology size is much larger. For Anatomy, reduction anchors–based approach does not demonstrate distinct advantages with the middle-size ontologies. However, for the track of FMA-NCI, especially for FMA-SNOMED, the runtime has been notably reduced compared with the other matchers. Besides, reduction anchors barely bring metric loss while simultaneously promoting the efficiency of matching. While integrating the reduction anchors together, it is effective to skip large numbers of ignorable similarity computations and is efficient to reduce the time complexity.

Table 10 presents the comparison of LOM-RAE with LOM-RA, which demonstrates the superiority of our extended reduction anchors compared with previous reduction anchors. From Table 10, we can observe that RAE is effective to skip much more similarity computations than RA. Because the similarity threshold is set properly high and the reduction set is obtained through strictly abiding by the defined constraints, it is evident there is almost little or no loss in performance with considerable matching comparisons being omitted.

In addition, to examine the practicability of the extended reduction anchors, we compare our matching approach based on RAE with some other systems participating in OAEI, for example, AML [16], LogMap [35], Wiktionary [54], and ALOD2Vec [55]. The execution times of these systems are shown in Table 11. In contrast to the matching systems utilizing modulization and clustering, such as AML and LogMap, the reduction set is generated dynamically based on the similarity calculations of entity pairs, which require much more time during matching. Nevertheless, it can be observed that our proposed approach can still achieve promising performance among these matching systems, which demonstrates that RAE is practicable and effective for large-scale ontology matching scenario.

**Table 9:** Effectiveness of extended reduction anchors.^a,b^

Task and matcher		P (%)	R (%)	F1 (%)	Time (minutes)
**Anatomy**					
	Term	92.28	74.14	82.22	1.5
	Term + Struc	91.82	81.40	86.30	3.0
	Term + Ext	92.67	88.46	90.52	4.9
	Term + Rps	93.31	74.39	83.02	2.8
	Term + Struc + Ext	93.78	90.44	92.08	7.1
	Term + Struc + Ext + Rps	**94.95**	**90.57**	**92.71**	8.9
	*Term + Struc + Ext + RAE*	94.74	90.36	92.50	**0.7**
**FMA^c^-NCI^d^**					
	Term	90.01	70.90	79.32	11.4
	Term + Struc	89.36	73.87	80.88	47.9
	Term + Ext	92.67	**88.46**	**90.52**	18.6
	Term + Rps	**93.31**	74.39	83.02	12.8
	Term + Struc + Ext	89.65	76.92	82.80	56.8
	Term + Struc + Ext + Rps	90.04	76.71	82.84	65.9
	*Term + Struc + Ext + RAE*	89.65	75.56	82.00	**2.8**
**FMA-SNOMED**					
	Term	35.49	36.59	36.03	64.4
	Term + Struc	37.21	41.59	39.27	85.6
	Term + Ext	46.78	52.60	49.52	113.4
	Term + Rps	**93.31**	**74.39**	**83.02**	117.1
	Term + Struc + Ext	47.97	52.21	50.00	161.0
	Term + Struc + Ext + Rps	47.52	52.01	49.66	227.5
	*Term + Struc + Ext + RAE*	53.41	51.25	52.31	**12.5**

^a^The best performing matcher is italicized.

^b^Values in bold indicate best experimental results.

^c^FMA: Foundation Model of Anatomy.

^d^NCI: National Cancer Institute.

**Table 10:** Effectiveness of extended reduction anchors.^a^

Task and matcher		P (%)	R (%)	F1 (%)	Time (minutes)
**Anatomy**					
	LOM-RA	*95.41*	*90.64*	*92.96*	2.2
	LOM-RAE	94.28	90.36	92.50	*0.7*
**FMA^b^-NCI^c^**					
	LOM-RA	*90.01*	*75.61*	*82.18*	10.4
	LOM-RAE	89.65	75.56	82.00	*2.8*
**FMA-SNOMED**					
	LOM-RA	*53.77*	50.89	52.29	42.7
	LOM-RAE	53.41	*51.25*	*52.31*	*12.5*

^a^Italicized values indicate best experimental results.

^b^FMA: Foundation Model of Anatomy.

^c^NCI: National Cancer Institute.

**Table 11:** Execution time (minutes) of systems.^a^

Matching system	Anatomy	FMA^b^-NCI^c^	FMA-SNOMED
AML	0.48	0.63	1.68
LogMap	0.01	0.03	0.87
Wikitionary	1.08	4.3	11.62
ALOD2Vec	3.93	2.97	—
LOM-RAE	*0.7*	*2.8*	*12.5*

^a^Italicized values indicate best experimental results.

^b^FMA: Foundation Model of Anatomy.

^c^NCI: National Cancer Institute.

### Performance of Extended Reduction Anchors

There is a need to analyze the influence of key parameters of our proposed reduction anchors. Here we use a new metric called benefit rate (*G*) to measure how much an LOM algorithm can improve the performance: *G* = *N*/(*n*_1_ * *n*_2_), where *N* is the size of the total reduction set; and *n*_1_ and *n*_2_ represent the number of concepts in 2 ontologies. The larger the value of *G*, fewer the times of similarity calculations required and the higher the efficiency of the algorithm.

**Figure 4:**
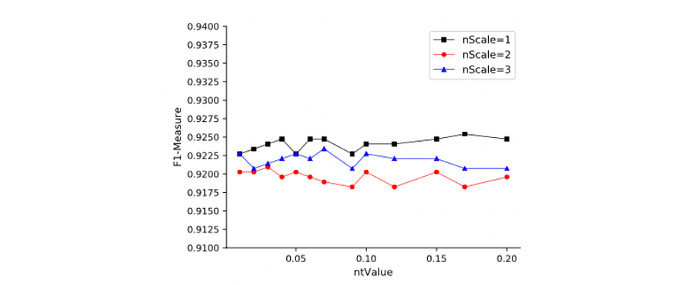
ntValue-nScale-matching quality.

**Figure 5:**
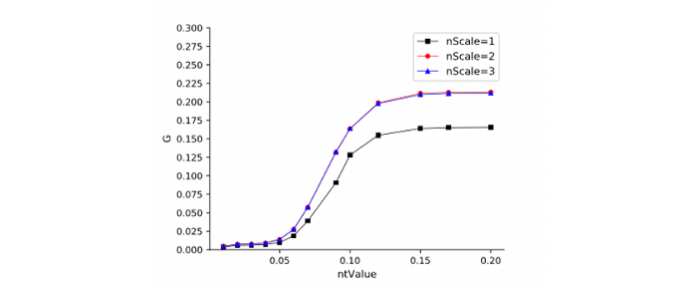
ntValue-nScale-benefit rate.

**Figure 6:**
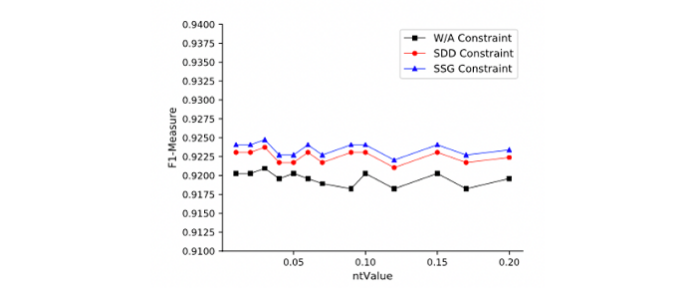
SDD-SSG-matching quality.

**Figure 7:**
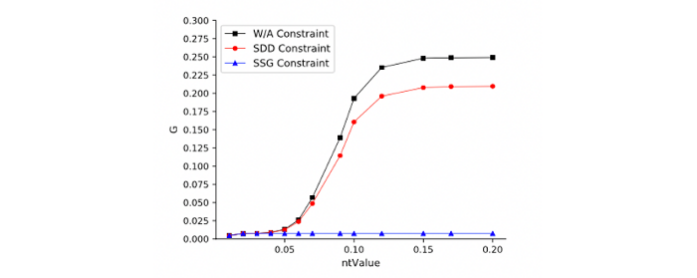
SDD-SSG-benefit rate.

The LOM-NE algorithm has 4 important parameters: *ntValue*, *nScale*, SDD constraint, and SSG constraint. We evaluate these parameters on the Anatomy data set. Figure 4 shows the relation between *ntValue* and F1 measure on different *nScales*. Figure 5 shows the relation between benefit rate and *ntValue* under different *nScales*. We observe that (1) *ntValue* has a certain effect on matching quality and efficiency, that is, different *ntValues* will lead to some fluctuation of matching quality. Meanwhile, LOM-NE also causes a higher benefit rate with an increase of *ntValue*. (2) *nScale* also affects matching quality and efficiency. As *nScale* increases, matching quality will decrease, but the benefit rate will increase to a certain extent. Results also show that *ntValue* = 0.15 and *nScale* = 3 will lead to a good matching quality and benefit rate. Figures 6 and 7 show the influences of SDD and SSG on matching quality and benefit rate. The W/A constraint represents the results without any constraint. We can see that (1) under 3 constraints, the matching quality will increase, but the benefit rate will decrease; (2) the SDD constraint has a higher influence on matching quality and benefit rate.

## Discussion

### Principal Findings

In this section, we discuss our experimental results according to the research questions. First, we will analyze the influence of the matchers in a single dimension. Second, we will report on how information imposes an effect on the final performance compared with the distinguishing clues of concepts. Finally, we will illustrate the practical benefits of adopting reduction anchors in large biomedical ontology matching.

### How Do the Different Combination Strategies of the Clues Perform in Ontology Matching?

After analyzing the results of matchers in 4 predefined dimensions separately, it is obvious that there are some parts playing an inessential role in the matching process. According to the results presented in Table 5, it can be observed that property degrades the performance in the Anatomy track, and using the transitive rule in hierarchy to gain more structural presentations would also input noise to mappings. For all these 3 tracks, the structural clues play a significant role in the matching process and bring about a positive improvement. While using external knowledge as auxiliary resources, the general dictionary, such as WordNet, has resulted in a less positive impact than the biomedical lexical, such as UBERON, as illustrated in Table 6. WordNet is a dictionary that works in the general domain, and may be deficient in synonymy for biomedical concepts or generate erroneous synonymy.

When one integrates the semantic embedding method into biomedical ontology matching, results from Table 7 suggest that despite the ability of the former to capture the underlying potential semantic, it can also worsen the results. Besides, the BERT model used in the common domain is incapable of catching the semantic in biomedicine (Table 7). The fine-tuned BioBERT model trained on data sets related to the test suite is much more competent than BioBERT. Therefore, mining and combining the key clues from ontologies and auxiliary sources are more important compared with utilizing the whole sources.

### How Effective Are the Combinations of Matchers Implemented in Ontology Matching?

According to the results illustrated in Table 8, the combination strategies of matcher have different levels of impact on the tracks. It is evident that although large amounts of information could be mined from ontologies, some may result in scarce improvement and bring about an increase in time complexity at the same time. It can be observed that incorporating the structural matcher and the external matcher could have momentous benefits to biomedical ontology matching. The representation learning matcher is able to boost the performance of Anatomy and FMA-BCI to some extent, but it causes a decline in the performance of FMA-SNOMED. As a result, combining all matchers is not helpful to promote mappings. Thus, for different tasks, matchers may exert different effects, either positive or negative.

### What Is the Performance of the Proposed Reduction Anchors While Matching Large Biomedical Ontologies?

The results listed in Tables 9-11 demonstrate that reduction anchors are effective in reducing the running time during large ontology matching, with the superiority becoming more comparative when the volume of ontology is much larger. Reduction sets leverage the hierarchy concept to skip subsequent matching between subconcepts of one concept and super-concepts of the other concept, which also include the extended highly related concept nodes. By contrast, if 2 concepts have low similarity, based on the locality phenomenon of matching, it can skip subsequent matching between 1 concept and the neighbors of the other concept as well as the concepts with high similarity. When the ontology is large, the structure of ontology graph becomes complicated which would possess deeper hierarchical levels. Therefore, extended reduction anchors are able to and practicable to skip more unnecessary computations.

### Conclusions

In this paper, we presented an empirical study of biomedical ontology matching based on a number of experiments performed on terminology-based, structure-based, external knowledge–based, representation learning–based measures in detail. Biomedical ontology matching relying on the terminological description of elements, combined with a structural, external knowledge, and embedding similarity approach, is effective for the matching of ontologies to some extent. According to our results, composite matchers are very effective. Despite the imprecision of single matchers, their combinations are impressive in improving the mapping quality, and bring about more accurate and stable similarity for biomedical ontologies. Structural and external clues are proved to produce better match results and support good precision as they could best compensate the shortcomings of single terminological matchers.

We can also find that the knowledge information has either a neutral or a negative impact on the F measure (as shown in Tables 4-7), which suggests that this result is an artifact. It is obvious that utilizing all the clues cannot always achieve best performance in ontology matching. The hierarchical interpretations play an important role in the matching task of Anatomy, whereas in FMA-NCI, they exert little influence, which relies rather much on terminologies. Using the WordNet dictionary to retrieve alternative labels for concepts only has a weak effect compared with domain-specific resources UBERON and UMLS. Furthermore, representation learning techniques are relatively effective to improve recall. However, it still needs to be further deepened and enhanced. Based on the results produced in the experiments, we can learn that utilizing credible and distinguishable clues can effectively boost the ontology matching process as compared with matching 2 ontologies with all.

Moreover, we propose a new, efficient, large ontology matching method based on extended reduction anchors. The proposed approach is generic and could be applied to different fields. RAE is applied to predict the ignorable similarity calculations in ontology matching. Our experimental results also overwhelmingly demonstrate that the proposed method presents significant and encouraging improvement, especially in runtime efficiency.

## Appendix

Multimedia Appendix 1 Algorithm 1.

Multimedia Appendix 2 Algorithm 2.

## Abbreviations

BERT: Bidirectional Encoder Representations from Transformers
BIC: Bayesian information criterion
FMA: Foundation Model of Anatomy
MELT: Matching EvaLuation Toolkit
NCI: National Cancer Institute
OAEI: Ontology Alignment Evaluation Initiative
OWL: Ontology Web Language
RDF: Resource Description Framework
RDFS: Resource Description Framework Schema
TF-IDF: term frequency-inverse document frequency
URI: uniform resource identifier
