# Supplementary material for: Matching Biomedical Ontologies: Construction of Matching Clues and Systematic Evaluation of Different Combinations of Matchers
Source: JMIR Med Inform. 2021 Aug 19;9(8):e28212. doi: 10.2196/28212 (PMC8414291; doi:10.2196/28212)
Supplement: Multimedia Appendix 1 [file medinform_v9i8e28212_app1.pdf]

Algorithm 1. LOM-PE algorithm

```
Input: ontology O1, ontology O2
Output: matching results
Function LOMPE_Algorithm(O1, O2)
begin
  │ foreach Li ∈ O1 do
  │   │ ComputeSim(Li)
  │ end
end
Function ComputeSim(L = (a1, a2,…, an))
begin
  │ PAE ← GetPAnchorsE(n/2)
  │ PSE ← PredictNewPSE(PAE)
  │ ComputeSim(La = (a1, a2,…, a(n/2)-1))
  │ ComputeSim(Lb = (a((n/2)+1),…, an)
  │ if |L| ≤ 1 then
  │   │ return
  │ end
end
Function GetP AnchorsE(ai)
begin
  │ foreach bj ∈ O2 do
  │   │ if (ai, bj) ∈ PSE then
  │   │   │ continue
  │   │ end
  │   │ Sim(ai, bj) ← Compute(ai, bj)
  │   │ if Sim(ai, bj) > ptValue then
  │   │   │ PAECandi ← PAECandi ∪ bj
  │   │ end
  │ end
  │ PAE ← MaxTopk(PAECandi)
end
```
