# Supplementary material for: Matching Biomedical Ontologies: Construction of Matching Clues and Systematic Evaluation of Different Combinations of Matchers
Source: JMIR Med Inform. 2021 Aug 19;9(8):e28212. doi: 10.2196/28212 (PMC8414291; doi:10.2196/28212)
Supplement: Multimedia Appendix 2 [file medinform_v9i8e28212_app2.pdf]

Algorithm 2: LOM-NE algorithm

```
Input: ontology O1, ontology O2
Output: matching results
begin
    SortConceptByDegree()
    foreach Ci ∈ O1 do
        NA(Ci) ← ∅
        foreach Dj ∈ O2 do
            if (Ci, Dj) ∈ NSE then
                continue
                s ←ComputeSim(Ci, Dj)
                if s < ntValueAND InConstraint() then
                    N AE(Ci) ← Dj
                end
                NSE(Ci) ← BuiltNSE(NAE(Ci))
                NSE(Ci) ← RefineNSE(NSE(Ci))
                NSE ← NSE ∪ NSE(Ci)
            end
        end
    end
end
```
